# CD133 expression is an independent prognostic marker for low survival in colorectal cancer

**DOI:** 10.1038/sj.bjc.6604664

**Published:** 2008-09-09

**Authors:** D Horst, L Kriegl, J Engel, T Kirchner, A Jung

**Affiliations:** 1 Pathologisches Institut der Ludwig-Maximilians-Universität München, Thalkirchner Str. 36, 80337 München, Germany; 2 Tumorregister München, Institut für medizinische Informationsverarbeitung, Biometrie und Epidemiologie der Ludwig-Maximilians-Universität München, Marchioninistr. 15, 81377 München, Germany

**Keywords:** CD133, colorectal, immunohistochemistry, prognosis, survival

## Abstract

Colon cancer cells have previously been demonstrated to contain a subpopulation of CD133+ tumour cells that have the ability to initiate tumour growth and are thus referred to as colon cancer-initiating cells or colon cancer stem cells (CSCs). As CD133 is currently one of the best markers to characterise colon CSCs, we analysed CD133+ tumour cells in colorectal cancer specimens using immunohistochemistry. We show that CD133 detection is specific and that the CD133 antigen is localised on the glandular-luminal surface of colon cancer cells, whereas undifferentiated tumour cells at the front of invasion are CD133−. In addition, CD133+ cells are characterised *in situ* by lack of CK20 expression, whereas they are positive for EpCAM. Moreover, we show that CD133 expression in colorectal cancer is an independent prognostic marker that correlates with low survival in a stratified patient collective. Our results indicate that in colorectal cancer, the CD133+ tumour cells can be detected by immunohistochemistry, which facilitates their further characterisation *in situ*.

The cancer stem cell (CSC) model is a current focus of cancer research. According to this model, cancer cannot be viewed as simple monoclonal expansions of functionally equal tumour cells. Instead, despite their monoclonal origin, only a small minority of tumour cells, the CSCs or ‘tumour-initiating cells’, have the ability to maintain the malignant population (Reya *et al*, 2001; Brabletz *et al*, 2005; Burkert *et al*, 2006).

Recent research findings suggest that among several other solid malignancies, the CSC model also applies for colon carcinoma. In this tumour, a CD133+ subpopulation of colon cancer cells was recently demonstrated to be highly enriched in tumour-initiating colon CSCs (Co-CSCs) that have the ability to self-renew and to recapitulate the bulk tumour population (O'Brien *et al*, 2007; Ricci-Vitiani *et al*, 2007). Furthermore, the CD133+ cells were able to retain tumorigenicity *in vitro* as spheroid cultures and were shown to be resistant to chemotherapeutic drugs, both characteristics of CSCs, whereas CD133− cells had none of these features (Ricci-Vitiani *et al*, 2007; Todaro *et al*, 2007). Thus, CD133 is currently one of the best markers for the characterisation of Co-CSCs.

CD133 is a five-transmembrane domain molecule with a molecular weight of 120 kDa (Miraglia *et al*, 1997). It has been shown to be located in apical plasma membrane protrusions of embryonal epithelial structures as well as on the cultured colon cancer cell line Caco-2 (Corbeil *et al*, 2000). It is implicated to mark stem-like cells of various tissues and cancers (reviewed in Mizrak *et al*, 2008). In colon cancer, CD133+ cells were shown to be devoid of the intestinal epithelial differentiation marker cytokeratin 20 (CK20), whereas they keep positivity for the epithelial adhesion molecule EpCAM (Ricci-Vitiani *et al*, 2007). Thus, in addition to their enrichment in Co-CSCs, CD133+ cells show further distinct surface characteristics as compared with CD133− tumour cells.

Although CD133 appears to be one of the most promising markers to isolate and further characterise Co-CSCs, nothing is known about the *in situ* localisation of CD133+ cells, their immunohistochemical features, or their relevance for patient outcome. Therefore, we investigated the expression of CD133 in colorectal cancer specimens of a highly stratified patient collective. We demonstrate that a subpopulation of tumour cells without the expression of the differentiation marker CK20 displays strong expression of CD133. This expression correlates significantly with patient outcome. Our findings support the importance of CD133+ tumour cells, and thus Co-CSCs for the malignant progression of colorectal cancer.

## Materials and methods

### Clinical samples

Cases were selected from colorectal cancer specimens of patients who underwent intentionally curative surgical resection between 1994 and 2004 at the Ludwig-Maximilians-Universität München. Follow up data were available from the Tumorregister München. Only moderately differentiated colorectal adenocarcinomas (G2 according to World Health Organization) with T-categories T2 or T3 and no nodal or distant metastasis at the time of diagnosis (N0, M0) were considered. To reduce effects, directly related to surgery, specimens of patients who died within 6 months after surgical resection were excluded. The final case collection included colorectal cancer specimens of 77 patients; of whom, 21 (27%) died of the diagnosed colorectal cancer within 5 years. The survival data of 47 cases (61%) were censored, as case follow up was discontinued or patients died of reasons other than colorectal cancer. In the final case collection, only the T-category (T2 *vs* T3), but neither age nor gender, was significantly associated with patient outcome. Case characteristics are summarised in Supplementary Table 1. The study complied with the guidelines of the local ethics committee.

### Immunohistochemistry

Immunohistochemical staining was carried out on 5 *μ*m sections of formalin-fixed, paraffin-embedded tumour samples. Comparative studies of CD133, CK20, and EpCAM were performed on adjacent serial sections of 10 tumours with moderate-to-high CD133 expression. As primary antibodies for CD133 (AC133 antigen, CD133/1) detection, we used anti-CD133 rabbit monoclonal antibody (mAb) (1 : 100, clone C24B9, Cell Signaling, Danvers, MA, USA), anti-CD133 mouse mAb (1 : 40, clone AC133, Miltenyi Biotech, Bergisch Gladbach, Germany), and anti-CD133 goat polyclonal antibody (1 : 200, Santa Cruz Biotechnology, Santa Cruz, CA, USA), the first of which was used for analysis of the patient samples. Further primary antibodies were anti-Ki67 mouse mAb (1 : 50, clone MIB-1, Dako, Glostrup, Denmark), anti-EpCAM mouse mAb (1 : 500, clone Ber-EP4, Dako), and anti-CK20 mouse mAb (1 : 300, clone Ks20.8, Progen Biotechnik, Heidelberg, Germany). For mouse and goat CD133 antibody staining, endogenous peroxidase was blocked using 7.5% hydrogen peroxide for 10 min and primary antibody incubation was carried out at room temperature for 60 min. For detection, the Vectastain ABC-Kits Elite Universal and Goat (Vector Laboratories, Burlingame, CA, USA) were used, respectively, and slides were developed with DAB (Dako). For all remaining antibodies, staining was performed on a Ventana Benchmark autostainer with the XT ultraView DAB Kit (Ventana Medical Systems, Illkirch, France). All slides were counterstained with Hematoxylin (Vector Laboratories). To exclude unspecific staining, isotype and system controls were included for each of the used antibodies.

### Evaluation of CD133 immunohistochemistry

CD133 immunostaining was evaluated on whole standard tissue sections of colorectal adenocarcinomas. Five medium power fields per section were viewed and tumours were given a semi-quantitative score of 0, <50, and ⩾50% CD133+ tumour glands. Positivity of glands was defined as either membranous staining or staining of shed cellular debris in the tumour glands. The specimens were evaluated by two pathologists who had no knowledge of prognosis and other clinicopathological variables.

### Western blotting

Whole protein lysates of near confluent Caco-2 and DLD-1 cultured cell lines were used. Cell lines were obtained from the American Type Culture Collection. A total of 45 *μ*g of protein were used per lane. Primary antibodies were anti-*β*-actin mouse mAb (1 : 10 000; Sigma-Aldrich, München, Germany), and the anti-CD133 antibodies that were used for immunohistochemistry (goat-anti-CD133 at 1 : 200; mouse-anti-CD133 at 1 : 200; and rabbit-anti-CD133 at 1 : 1000). Secondary antibodies were horseradish peroxidase-coupled anti-rabbit, anti-mouse (both GE Healthcare Europe, Freiburg, Germany), and anti-goat (Thermo Scientific, Bonn, Germany). Enhanced chemoluminescence (Amersham Biosciences Europe, Freiburg, Germany) was used for detection, according to the manufacturer's protocol.

### Statistical analysis

Cross-tabulations were calculated using Fisher's exact test. The Kaplan–Meier analysis was used to estimate cancer-specific survival, and the groups were compared with the log-rank test. Multivariate analysis was based on the Cox regression model. Statistical procedures were performed using SPSS version 15.0 (SPSS Inc., Chicago, IL, USA). *P*<0.05 was considered statistically significant.

## Results

### CD133 is specifically detected in colorectal cancer specimens by immunohistochemistry

**Figure 1:**
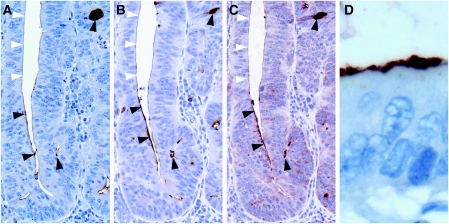
CD133 expression is found on the glandular-luminal surface of epithelial tumour cells. Three different antibodies were compared on serial tumour sections: (**A**) monoclonal anti-CD133 (Cell Signalling), (**B**) monoclonal anti-CD133 (Miltenyi Biotech), and (**C**) polyclonal anti-CD133 (Santa Cruz Biotechnology). All three antibodies showed comparable staining patterns. Arrowheads indicate positive (black) and negative (white) staining; original magnification, × 200. (**D**) High magnification of positively stained tumour cells; original magnification, × 630.

**Figure 2:**
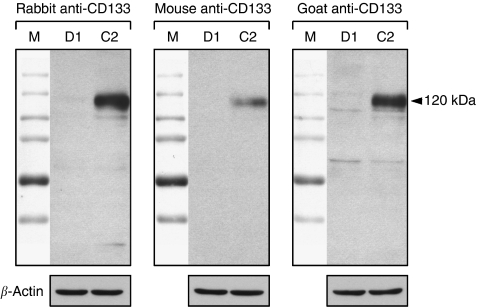
Specificity of CD133 detection. Protein lysates of the CD133− DLD-1 (D1) and the CD133+ Caco-2 (C2) colon cancer cell lines were blotted and stained with the three anti-CD133 antibodies used for immunohistochemistry. All three antibodies strongly detected the CD133 antigen in Caco-2, displayed at 120 kDa, whereas DLD-1 showed no staining. Unspecific bands that were observed were considered to be negligibly weak. *β*-Actin was used as control. Upper panel images show full-length Western blots merged with their molecular weight markers (M).

Before colorectal cancer specimens were immunohistochemically evaluated for CD133 expression, specificity of the antibodies had to be determined. Therefore, we compared serial tumour sections stained with three different antibodies raised against CD133. Those included the antibody that was previously used for the isolation of CD133+ cells from tumour cell suspensions (O'Brien *et al*, 2007; Ricci-Vitiani *et al*, 2007). All three antibodies revealed comparable staining patterns of positively and negatively stained tumour cells (Figure 1A–C). In addition, Western analysis of cell lysates from the CD133+ colon cancer cell line Caco-2 (Corbeil *et al*, 2000) and the CD133− cell line DLD-1 (unpublished results) showed specific protein detection with all three used antibodies (Figure 2). Therefore, the immunohistochemical *in situ* approach specifically detects the CD133 antigen.

### The CD133 antigen is located at the glandular-luminal surface of colorectal cancer cells

**Figure 3:**
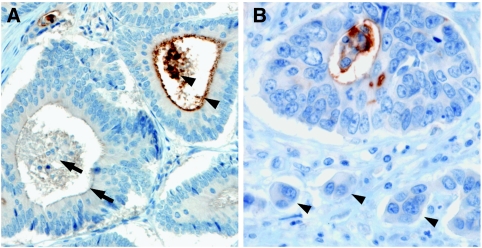
(**A**) CD133 staining of shed intraglandular cellular debris mirrors CD133 expression of the surrounding tumour cells. Arrows indicate a CD133− tumour gland filled with CD133− debris. Arrowheads indicate a CD133+ gland with CD133+ cellular debris and apical staining of the tumour cells. Tumour glands were considered as CD133+ if either apical CD133 staining of the tumour cells or CD133+ cellular debris or both were present. (**B**) CD133 only marks tumour cells that are gland forming. Undifferentiated tumour ‘buds’ at the front of tumour invasion are CD133− (arrowheads). Original magnification, × 200 (**A**), × 400 (**B**).

The CD133 antigen has previously been shown to be located in apical plasma membrane protrusions of embryonic epithelial cells as well as on the apical cell surface of the cultured colon cancer cell line Caco-2 (Corbeil *et al*, 2000). Comparably, we observed staining of CD133 on the luminal cell surface of colorectal cancer glands (Figure 1D). Only tumour cells with direct contact to these luminal surfaces were CD133+. Usually, several CD133+ tumour cells were grouped together with some glands being completely positive. In addition, most CD133-expressing tumours showed shedding of CD133+ cellular debris into the glandular lumina (Figure 3A). In tumour glands with CD133+ tumour cells, intraglandular cellular debris was always, at least partially, CD133+. When intraglandular debris was CD133−, the tumour cells were always CD133−. CD133 expression tended to be pronounced close to the invasive margin of the tumours (Figure 3B). However, small groups of infiltrating tumour cells at the front of tumour invasion, referred to as tumour ‘buds’ (Hase *et al*, 1993), showing no lumen formation, were generally CD133− (Figure 3B). Taken together, CD133 expression in colorectal cancer is polar, confined to the apical luminal surface of colorectal cancer cells with glandular differentiation, which is in accordance with the previous results. In addition, CD133 staining of intraglandular cellular debris mirrors CD133 expression of the surrounding tumour cells.

### CD133+ colorectal cancer cells lack CK20 expression while they are EpCAM+

**Figure 4:**
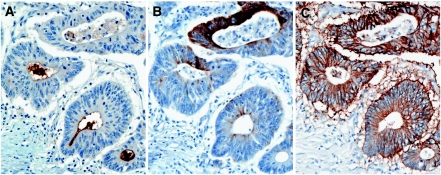
CD133 and CK20 expressions are mutually exclusive. Serial sections stained for CD133 (**A**), CK20 (**B**), and EpCAM (**C**) demonstrate that CD133+ cells are negative for CK20, whereas CD133+ and CD133− cells express the EpCAM antigen. Original magnification, × 200.

Previous studies using cell suspensions of colon cancer specimens demonstrated that CD133+ cells were CK20−, whereas both CD133+ as well as CD133− cells expressed the epithelial cell adhesion molecule EpCAM (Ricci-Vitiani *et al*, 2007). Thus, we expected to find comparable results on colorectal cancer cells *in situ*. Therefore, we used serial tumour sections and stained for CD133, CK20, and EpCAM. Expectedly, areas with many CD133+ cells showed none to very low CK20 expression, whereas areas expressing high amounts of CK20 were generally CD133− (Figure 4). Furthermore, the tumours contained subpopulations of cells that expressed neither CD133 nor CK20. EpCAM was detected in comparable amounts in both CD133+ and CD133− areas (Figure 4). These patterns were found in 8 out of 10 tumour cases, whereas 2 cases were not evaluable as they showed widely distributed presence of CD133, which did not allow a reliable correlation with CK20 staining (data not shown). The expression pattern of our *in situ* results are thus in line with previously published data about the CD133+ cell phenotype.

### CD133 expression in colorectal cancer strongly correlates with patient survival

**Figure 5:**
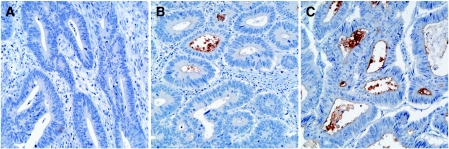
Scoring of CD133 expression in colorectal cancer. Tumours that showed no staining (**A**) and tumours with less than 50% positively stained glands (**B**) were considered as CD133-low; tumours with more than 50% positively stained glands as CD133-high (**C**). Original magnification, × 200.

**Figure 6:**
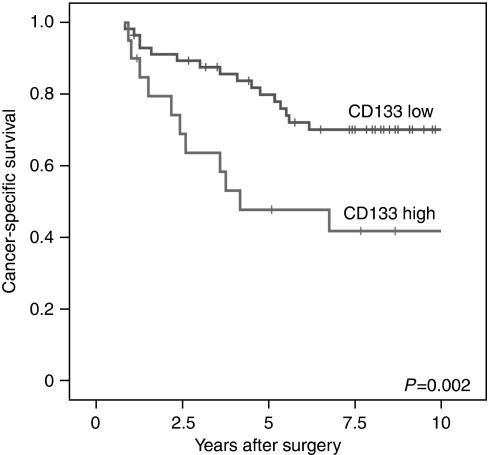
CD133 expression correlates with low survival. The Kaplan–Meier plot of colorectal cancer specimens (*n*=77) demonstrates significant (log-rank test) lower survival with CD133-high expression.

CD133 expression was scored on the basis of CD133+ tumour glands. Tumours showing more than 50% positive glands (either apical staining or staining of intraglandular cellular debris) were considered as CD133-high, whereas those with less than 50% stained glands were CD133-low (Figure 5). Applying this criterion, 26% of the tumours were CD133-high and 74% were CD133-low. When comparing the CD133 status with clinicopathological variables, no correlation with age, gender, or T-category of the tumour was observed (Table 1). Next, we examined the correlation of CD133 expression with patient outcome employing the Kaplan–Meier analysis. Patients with CD133-high tumours were associated with a significant worse 5- and 10-year survival than those with CD133-low tumours (*P*=0.002; Figure 6). Applying multivariate Cox regression, we found that the CD133-high expression represented an independent relative risk of 2.45 compared with the CD133-low group (*P*=0.018; Table 2). While age and gender were not significantly associated with outcome, the only other parameter that was marginally significant in multivariate analysis was the T-category with T3 having a relative risk of 2.51 compared with T2 (*P*=0.073).

## Discussion

In this study, we investigated the expression of CD133 in colorectal cancer using an immunohistochemical approach. As the localisation of CD133+ colorectal cancer cells has not yet been characterised *in situ*, we had to verify that our approach truly detects the CD133 antigen. Thus, we used three different antibodies that were specific on the protein level and showed comparable staining patterns on serial tumour sections. In addition, we demonstrate that CD133+ cell groups were CK20− and EpCAM+, which is in accordance with previous studies that characterised the phenotype of CD133+ colon cancer cells using tumour cell suspensions (Dalerba *et al*, 2007; Ricci-Vitiani *et al*, 2007). Moreover, we found the CD133 antigen at the luminal surface of epithelial tumour glands with shedding into the lumina, while no other staining pattern was observed. This complies with previous results of CD133 detection in embryonal tissue and on the apical surface of the cultured colon cancer cell line Caco-2 (Corbeil *et al*, 2000). Taken together, the specificity of our results is supported by previous studies in colorectal cancer. However, staining results, which differ from the positivity of the luminal surface in colorectal cancer, have recently been reported in pancreatic cancer, where a cytoplasmic expression of CD133 was shown (Hermann *et al*, 2007; Maeda *et al*, 2008). Such organ-specific patterns of CD133 expression still have to be clarified in further studies.

The CD133+ subpopulation of colon cancer cells was recently demonstrated to be highly enriched in tumour-initiating Co-CSCs, whereas in contrast, CD133− tumour cells did not have tumour-initiating capabilities (O'Brien *et al*, 2007; Ricci-Vitiani *et al*, 2007). CD133 is therefore currently one of the best markers for the detection of Co-CSCs. As Co-CSCs are hypothesised to be central for tumour relapse and progression (Brabletz *et al*, 2005; Ricci-Vitiani *et al*, 2008), we investigated CD133 expression in a collection of colorectal adenocarcinomas that was highly stratified towards patient outcome. Expectedly, CD133 expression was strongly associated with patient survival and proofed to be an independent prognostic marker.

Another prognostic marker that has previously been described for colon cancer is tumour ‘budding’; that is, small clusters of undifferentiated cells ahead of the invasive front (Hase *et al*, 1993; Ueno *et al*, 2002). We therefore expected to find CD133+ budding tumour cells in colorectal cancer. Although CD133 expression was pronounced in tumour glands close to the invasive margin, CD133 expression was restricted to glandular differentiated cells and budding tumour cells were generally CD133−. As both makers correlate with patient survival, it might be speculated whether budding tumour cells contain a population of Co-CSCs that are not characterised by CD133 expression.

Although CD133 is currently one of the best markers to characterise Co-CSCs, by far not every CD133+ cell is a Co-CSC. It was estimated that 1 in 262 CD133+ colon cancer cells actually has tumour-initiating capabilities (O'Brien *et al*, 2007). We demonstrate that in some tumours, several cells or even whole tumour glands are CD133+, whereas the actual Co-CSCs among them cannot be further confined using this marker. Thus, although the immunostaining for CD133 might correlate with the amount of Co-CSCs and can be used to estimate their impact on prognosis, CD133 appears to be inappropriate for the morphological characterisation of single Co-CSCs and their niche.

Taken together, we provide further evidence that the CD133+ tumour cell population in colorectal cancer is specifically, although not exclusively, important for colon cancer progression, and we present an approach that can be applied for further characterisation of these cells *in situ*.

## Figures and Tables

**Table 1:** CD133 expression does not correlate with age, gender, or T-category of the investigated colorectal cancer cases

**Variable**	**CD133 low**	**CD133 high**	***P*-value**
*Gender*			
Male	36	10	0.43
Female	21	10	
			
*Age, years*			
⩽69	31	11	1.0
⩾70	26	9	
			
*T-category*			
T2	24	4	0.11
T3	33	16	

**Table 2:** Multivariate survival analysis

**Variable**	**Relative risk (95% confidence interval)**	***P*-value**
*CD133*		
Low	1.00	
High	2.45 (1.18–5.13)	0.018
		
*Gender*		
Male	1.00	
Female	0.88 (0.42–1.84)	0.74
		
*Age, years*		
⩽69	1.00	
⩾70	1.28 (0.61–2.65)	0.52
		
*T-category*		
T2	1.00	
T3	2.51 (0.92–6.85)	0.073

CD133 expression in colorectal carcinoma is an independent marker for low patient survival.
