# Supplementary material for: CD133 expression is an independent prognostic marker for low survival in colorectal cancer
Source: Br J Cancer. 2008 Sep 9;99(8):1285–9. doi: 10.1038/sj.bjc.6604664 (PMC2570510; doi:10.1038/sj.bjc.6604664)
Supplement: Supplementary Table 1 [file 6604664x1.doc]

**Supplementary table 1.** Clinicopathological characteristics of the investigated colorectal cancer cases.

| **Variable** | **Number of cases** | **%** |
| --- | --- | --- |
| Gender |  |  |
| Male | 46 | 60 |
| Female | 31 | 40 |
| Age, y |  |  |
| ≤ 69 | 42 | 55 |
| ≥ 70 | 35 | 45 |
| T-category |  |  |
| T2 | 28 | 36 |
| T3 | 49 | 64 |
| Cancer specific survival, y |  |  |
| < 5 | 21 | 27 |
| ≥ 5 | 50 | 65 |
| Censored | 47 | 61 |
